# Design, Synthesis and Evaluation of Novel Isoxazolines/Oxime Sulfonates of 2′(2′,6′)-(Di)Chloropodophyllotoxins as Insecticidal Agents

**DOI:** 10.1038/srep33062

**Published:** 2016-09-26

**Authors:** Mingqiao Yu, Guangci Liu, Yuanyuan Zhang, Tao Feng, Ming Xu, Hui Xu

**Affiliations:** 1 Research Institute of Pesticidal Design & Synthesis, College of Sciences, Northwest A&F University, Yangling 712100, Shaanxi Province, P. R. China; 2 Shaanxi Key Laboratory of Natural Products & Chemical Biology, College of Plant Protection, Northwest A&F University, Yangling 712100, Shaanxi Province, P. R. China

## Abstract

A series of 2′(2′,6′)-(di)halogeno-isoxazolopodophyllic acids-based esters, and oxime sulfonates of 2′(2′,6′)-(di)halogenopodophyllones were prepared by structural modifications of podophyllotoxin as insecticidal agents against *Mythimna separata* Walker. It was found that when 2′(2′,6′)-(di)halogenopodophyllones or 2′(2′,6′)-(di)chloropicropodophyllones reacted with hydroxylamine hydrochloride, the desired products were related with the configuration of their lactones. Three key single-crystal structures of Ie, IIe and IIIb were determined by X-ray diffraction. Especially compounds IIc and Vc showed the highest insecticidal activity. Moreover, some interesting results of structure-insecticidal activity relationships of tested compounds were also observed.

The oriental armyworm, *Mythimna separata* (Walker) (Lepidoptera: Noctuidae), a typical and key agricultural insect pest in China, Korea, Japan, Southeast Asia, Australia and Oceania, could extensively damage important cereal crops such as maize, wheat and rice^1^,^2^. Currently, chemical control is still an essential component of crop protection in modern agriculture, and a variety of synthetic agrochemicals have been widely used to manage insect pests. However, a wide range of application of those agrochemicals has resulted in ecological disturbances and increasing resistance in pest populations, which will make the species more difficult to control^3^,^4^,^5^,^6^,^7^,^8^. Therefore, it is necessary to develop new potential alternatives to effectively and selectively control insect pests in the agricultural field^4^,^5^,^6^,^7^,^8^. In recent decades, the discovery and development of new pesticides directly or indirectly from plant secondary metabolites has been received much attention due to their less or slower resistance development and lower environmental pollution^9^,^10^,^11^,^12^,^13^,^14^,^15^,^16^.

Podophyllotoxin (**1**, Fig. 1), a naturally occurring cyclolignan, is isolated from the roots and rhizomes of some *Podophyllum* and *Juniperus* species. Compound **1** has been used as the lead compound for preparation of three clinical anticancer drugs such as etoposide (VP-16), teniposide (VM-26) and etoposide phosphate. Additionally, compound **1** also exhibits other interesting properties such as cytotoxic, insecticidal, antifungal, and antiviral activities^17^. Therefore, total synthesis^18^,^19^,^20^ and structural modification^21^,^22^,^23^ of **1** and its analogs is always the research topic.

More recently, a series of oxime sulfonate derivatives of 2′(2′,6′)-(di)chloropicropodophyllotoxins (**2**, Fig. 1, Eq. (1)) have been synthesized and some compounds showed more potent insecticidal activity than toosendanin, a commercial botanical insecticide isolated from *Melia azedarach*^24^. Additionally, the *trans*-lactone was an important factor for podophyllotoxins exhibiting the good insecticidal activity^25^. Especially when a chlorine atom was introduced at the C-2′ position on the E-ring of podophyllotoxin derivatives, the corresponding compounds showed no significant cytotoxicity^26^. Based upon the above results, in the present paper we wanted to prepare a series of oxime sulfonate derivatives of 2′(2′,6′)-(di)chloropodophyllotoxins (**3**, Fig. 1, Eq. (2)). Their insecticidal activity tested against a typical lepidopteran pest (*M. separata*) was also presented.

## Methods

### Materials and Instruments

Podophyllotoxin was purchased from Gansu Gerui Medicinal Materials Co., Ltd. (Lanzhou, China). All chemical reagents were purchased and utilized without further purification. Solvents were used directly or treated with standard methods before use. Analytical thin-layer chromatography (TLC) and preparative thin-layer chromatography (PTLC) were performed with silica gel plates using silica gel 60 GF254 (Qingdao Haiyang Chemical Co., Ltd., Qingdao, China). Silica gel column chromatography was performed with silica gel 200–300 mesh (Qingdao Haiyang Chemical Co., Ltd., Qingdao, China). Melting points (mp) were determined on a XT-4 digital melting point apparatus. Optical rotation was measured on a Rudolph Research Analytical Autopol III automatic polarimeter. Infrared spectra (IR) were recorded on a Bruker TENSOR 27 spectrometer. Proton nuclear magnetic resonance spectra (^1^H NMR) were recorded in CDCl_3_ on a Bruker Avance III 500 MHz instrument using tetramethylsilane (TMS) as the internal standard. High-resolution mass spectra (HR-MS) were carried out with IonSpec 4.7 Tesla FTMS instrument.

### General procedure for synthesis of 2′(2′,6′)-(di)halogenopodophyllones (7–9)

A mixture of 2′(2′,6′)-(di)halogenopodophyllotoxin (**4**, **5**, or **6**, 1 mmol), CrO_3_ (5 mmol), and pyridine (10 mmol) in dry dichloromethane (DCM, 20 mL) was stirred at room temperature. When the reaction was complete checked by TLC analysis, the mixture was diluted by DCM (60 mL), washed by saturated aq. NaHSO_3_ (30 mL) and brine (30 mL), dried over anhydrous Na_2_SO_4_, concentrated under reduced pressure, and purified by silica gel column chromatography eluting with petroleum ether/ethyl acetate (2:3, v/v) to afford compounds **7**–**9**.

*Data for* **7:** CAS: 1458601-16-5. Yield = 81%, white solid, m.p. 217–218 °C [lit. 218–219 °C]^27^; [α]^20^_D_ = −64 (*c* 2.7 mg/mL, CHCl_3_); IR cm^−1^ (KBr): 3075, 2933, 1787, 1685, 1487, 1391, 1117, 1020; ^1^H NMR (500 MHz, CDCl_3_) *δ*: 7.49 (s, 1H, H-5), 6.57 (s, 1H, H-8), 6.19 (s, 1H, H-6′), 6.05 (d, *J* = 3.0 Hz, 2H, OCH_2_O), 5.42 (d, *J* = 5.5 Hz, 1H, H-1), 4.58–4.62 (m, 1H, H-11), 4.33–4.36 (t, *J* = 9.5 Hz, 1H, H-11), 3.92 (s, 3H, OCH_3_), 3.88 (s, 3H, OCH_3_), 3.76–3.80 (m, 1H, H-3), 3.65 (s, 3H, OCH_3_), 3.40 (dd, *J* = 16.0, 5.5 Hz, 1H, H-2); HRMS (ESI): Calcd for C_22_H_19_ClO_8_Na ([M+Na]^+^) 469.0671, found 469.0660.

*Data for* **8:** Yield = 85%, white solid, m.p. 224–225 °C; [α]^20^_D_ = −80 (*c* 3.0 mg/mL, CHCl_3_); IR cm^−1^ (KBr): 3084, 2937, 1789, 1686, 1479, 1395, 1249, 1062; ^1^H NMR (500 MHz, CDCl_3_) *δ*: 7.47 (s, 1H, H-5), 6.49 (s, 1H, H-8), 6.05 (s, 2H, OCH_2_O), 5.73 (d, *J* = 7.5 Hz, 1H, H-1), 4.59–4.62 (m, 1H, H-11), 4.28–4.35 (m, 1H, H-11), 3.97 (s, 3H, OCH_3_), 3.96 (s, 3H, OCH_3_), 3.82–3.86 (m, 4H, H-3 and OCH_3_), 3.52–3.57 (m, 1H, H-2); HRMS *m/z* calcd for C_22_H_21_O_8_NCl ([M+H]^+^) 481.0451, found 481.0447.

*Data for* **9:** CAS: 37158-57-9. Yield = 83%, white solid, m.p. 222–223 °C [lit. 220–221 °C]^27^; [α]^20^_D_ = −93 (*c* 2.9 mg/mL, CHCl_3_); IR cm^−1^ (KBr): 3074, 2934, 1789, 1685, 1479, 1391, 1196, 1075; ^1^H NMR (500 MHz, CDCl_3_) *δ*: 7.50 (s, 1H, H-5), 6.57 (s, 1H, H-8), 6.18 (s, 1H, H-6′), 6.06 (d, *J* = 3.5 Hz, 2H, OCH_2_O), 5.52 (d, *J* = 4.5 Hz, 1H, H-1), 4.60–4.64 (m, 1H, H-11), 4.34–4.38 (m, 1H, H-11), 3.92 (s, 3H, OCH_3_), 3.88 (s, 3H, OCH_3_), 3.80–3.84 (m, 1H, H-3), 3.64 (s, 3H, OCH_3_), 3.40 (dd, *J* = 16.0, 5.5 Hz, 1H, H-2); HRMS (ESI): Calcd for C_22_H_19_BrO_8_Na ([M+Na]^+^) 513.0154, found 513.0155.

### General procedure for synthesis of 2′(2′,6′)-(di)halogeno-isoxazolopodophyllic acids (10–12) and oximes of 2′(2′,6′)-(di)halogenopodophyllones (13–15)

A mixture of 2′(2′,6′)-(di)halogenopodophyllone (**7**, **8** or **9**, 1 mmol), hydroxylamine hydrochloride (1.5 mmol), and pyridine (4 mmol) in absolute ethanol (20 mL) was refluxed. When the reaction was complete checked by TLC analysis, the solvent was removed under reduced pressure, and saturated aq. NaHCO_3_ (15 mL) was added to the residue, which was extracted with ethyl acetate (3 × 30 mL). The combined organic phase was dried over anhydrous Na_2_SO_4_, filtered, concentrated under reduced pressure, and purified by silica gel column chromatography eluting with DCM/methanol (98:2, v/v) to afford compounds **10–15**. For compounds **13–15** were not stable, they were used directly for the next step.

*Data for* **10**: Yield = 65%, white solid, m.p. 174–175 °C; [α]^20^_D_ = −78 (*c* 2.0 mg/mL, CHCl_3_); IR cm^−1^ (KBr): 3447, 2930, 1713, 1483, 1583, 1233, 1036; ^1^H NMR (500 MHz, CDCl_3_) *δ*: 7.43 (s, 1H, H-5), 6.52 (s, 1H, H-8), 6.07 (s, 1H, H-6′), 5.99 (d, *J *= 8.0 Hz, 2H, OCH_2_O), 5.35 (d, *J* = 5.5 Hz, 1H, H-1), 4.79 (t, *J* = 9.0 Hz, 1H, H-11), 4.01–4.09 (m, 1H, H-11), 3.87 (s, 3H, OCH_3_), 3.85 (s, 3H, OCH_3_), 3.79–3.84 (m, 1H, H-3), 3.62 (s, 3H, OCH_3_), 3.28 (dd, *J* = 13.0, 5.5 Hz, 1H, H-2); HRMS *m/z* calcd for C_22_H_21_O_8_NCl ([M+H]^+^) 462.0950, found 462.0943.

*Data for* **11**: Yield = 63%, white solid, m.p. 200–201 °C ; [α]^20^_D_ = −150 (*c* 2.4 mg/mL, CHCl_3_); IR cm^−1^ (KBr): 3437, 3108, 2938, 1711, 1480, 1230, 1096; ^1^H NMR (500 MHz, CDCl_3_) *δ*: 7.37 (s, 1H, H-5), 6.33 (s, 1H, H-8), 5.98 (d, *J* = 3.5 Hz, 2H, OCH_2_O), 5.72 (d, *J *= 8.5 Hz, 1H, H-1), 4.74–4.78 (m, 1H, H-11), 4.51–4.56 (m, 1H, H-11), 3.94 (s, 3H, OCH_3_), 3.89 (s, 3H, OCH_3_), 3.76–3.80 (m, 4H, H-3 and OCH_3_), 3.38 (dd, *J* = 13.0, 8.5 Hz, 1H, H-2); HRMS *m/z* calcd for C_22_H_20_O_8_NCl_2_ ([M+H]^+^) 496.0560, found 496.0553.

*Data for* **12**: Yield = 60%, white solid, m.p. 194–195 °C; [α]^20^_D_ = −109 (*c* 2.7 mg/mL, CHCl_3_); IR cm^−1^ (KBr): 3446, 3058, 2936.1709, 1482, 1234, 1105; ^1^H NMR (500 MHz, CDCl_3_) *δ*: 7.37 (s, 1H, H-5), 6.45 (s, 1H, H-8), 6.15 (s, 1H, H-6′), 5.98 (d, *J* = 16.5 Hz, 2H, OCH_2_O), 5.24 (d, *J* = 5.0 Hz, 1H, H-1), 4.73 (t, *J* = 8.5 Hz, 1H, H-11), 3.98–4.05 (m, 1H, H-11), 3.81 (s, 3H, OCH_3_), 3.80 (s, 3H, OCH_3_), 3.71–3.76 (m, 1H, H-3), 3.59 (s, 3H, OCH_3_), 3.03 (dd, *J* = 12.5, 6.0 Hz, 1H, H-2); HRMS *m/z* calcd for C_22_H_21_O_8_NBr ([M+H]^+^) 506.0445, found 506.0440.

### General procedure for synthesis of 2′(2′,6′)-(di)halogeno-isoxazolopodophyllic acids-based esters (Ia–c,e–g; IIa–f; and IIIa–g)

A mixture of the corresponding alcohols R^1^OH (0.28 mmol), *N, N*′-dicyclohexylcarbodiimide (DCC, 0.2 mmol), 4-dimethylaminopyridine (DMAP, 0.04 mmol), and 2′(2′,6′)-(di)halogeno-isoxazolopodophyllic acids (**10**, **11**, or **12**, 0.2 mmol) in dry DCM (10 mL) was stirred at room temperature. When the reaction was complete according to TLC analysis, the mixture was diluted by DCM (40 mL), washed by water (20 mL), aq. HCl (0.1 mol/L, 20 mL), saturated aq. NaHCO_3_ (20 mL) and brine (20 mL), dried over anhydrous Na_2_SO_4_, concentrated in vacuo, and purified by PTLC to give compounds **Ia–c,e–g; IIa–f;** and **IIIa–g** in 47–93% yields. The example data of **Ia–c; IIa–c;** and **IIIa–c** are listed as follows, whereas data of **Ie–g; IId–f;** and **IIId–g** can be found in the Supporting Information.

*Data for* **Ia**: Yield = 63%, white solid, m.p. 155–156 °C; [α]^20^_D_ = −81 (*c* 3.0 mg/mL, CHCl_3_); IR cm^−1^ (KBr): 3094, 2937, 1736, 1484, 1233, 1109; ^1^H NMR (500 MHz, CDCl_3_) *δ*: 7.43 (s, 1H, H-5), 6.50 (s, 1H, H-8), 6.08 (s, 1, H, H-6′), 5.99 (dd, *J* = 8.0, 1.5 Hz, 2H, OCH_2_O), 5.31 (d, *J* = 5.5 Hz, 1H, H-1), 4.78–4.81 (m, 1H, H-11), 4.06–4.13 (m, 1H, H-11), 3.89 (s, 3H, OCH_3_), 3.86 (s, 3H, OCH_3_), 3.76–3.80 (m, 1H, H-3), 3.62 (s, 3H, OCH_3_), 3.61 (s, 3H, CO_2_CH_3_), 3.27 (dd, *J* = 13.0, 5.5 Hz, 1H, H-2); HRMS *m/z* calcd for C_23_H_23_O_8_NCl ([M+H]^+^) 476.1107, found 476.1099.

*Data for* **Ib**: Yield = 54%, white solid, m.p. 129–130 °C, [α]^20^_D_ = −91 (*c* 3.0 mg/mL, CHCl_3_); IR cm^−1^ (KBr): 3036, 2931, 1728, 1484, 1232, 1110; ^1^H NMR (500 MHz, CDCl_3_) *δ*: 7.43 (s, 1H, H-5), 6.51 (s, 1H, H-8), 6.11 (s, 1H, H-6′), 5.99 (dd, *J* = 9.0, 1.0 Hz, 2H, OCH_2_O), 5.34 (d, *J* = 6.0 Hz, 1H, H-1), 4.79–4.82 (m, 1H, H-11), 4.07–4.15 (m, 2H, CO_2_C*H*_2_CH_3_), 3.94–3.97 (m, 1H, H-11), 3.89 (s, 3H, OCH_3_), 3.85 (s, 3H, OCH_3_), 3.77–3.81 (m, 1H, H-3), 3.63 (s, 3H, OCH_3_), 3.25 (dd, *J* = 13.0, 6.0 Hz, 1H, H-2), 1.19 (t, *J* = 7.0 Hz, 3H, CO_2_CH_2_C*H*_3_); HRMS *m/z* calcd for C_24_H_25_O_8_NCl ([M+H]^+^) 490.1263, found 490.1255.

*Data for* **Ic**: Yield = 50%, white solid, m.p. 102–103 °C, [α]^20^_D_ = −100 (*c* 3.1 mg/mL, CHCl_3_); IR cm^−1^ (KBr): 3105, 2938, 1728, 1483, 1226, 1111; ^1^H NMR (500 MHz, CDCl_3_) *δ*: 7.43 (s, 1H, H-5), 6.51 (s, 1H, H-8), 6.10 (s, 1H, H-6′), 5.99 (dd, *J* = 9.0, 1.0 Hz, 2H, OCH_2_O), 5.33 (d, *J *= 5.5 Hz, 1H, H-1), 4.77–4.81 (m, 1H, H-11), 4.10–4.14 (m, 1H, H-11), 4.02–4.09 (m, 2H, CO_2_C*H*_2_CH_2_CH_2_CH_3_), 3.89 (s, 3H, OCH_3_), 3.85 (s, 3H, OCH_3_), 3.77–3.81 (m, 1H, H-3), 3.64 (s, 3H, OCH_3_), 3.26 (dd, *J *= 12.5, 6.0 Hz, 1H, H-2), 1.47–1.58 (m, 2H, CO_2_CH_2_C*H*_2_CH_2_CH_3_), 1.27–1.32 (m, 2H, CO_2_CH_2_CH_2_C*H*_2_CH_3_), 0.91 (t, *J *= 7.5 Hz, 3H, CO_2_CH_2_CH_2_CH_2_C*H*_3_); HRMS *m/z* calcd for C_26_H_29_O_8_NCl ([M+H]^+^) 518.1576, found 518.1568.

*Data for* **IIa**: Yield = 71%, white solid, m.p. 113–114 °C; [α]^20^_D_ = −90 (*c* 2.7 mg/mL, CHCl_3_); IR cm^−1^ (KBr): 3078, 2935, 1734, 1483, 1230, 1109; ^1^H NMR (500 MHz, CDCl_3_) *δ*: 7.38 (s, 1H, H-5), 6.33 (s, 1H, H-8), 5.97 (dd, *J* = 4.0, 1.5 Hz, 2H, OCH_2_O), 5.70 (d, *J *= 9.0 Hz, 1H, H-1), 4.76–4.79 (m, 1H, H-11), 4.58–4.64 (m, 1H, H-11), 3.94 (s, 3H, OCH_3_), 3.91 (s, 3H, OCH_3_), 3.80 (s, 3 H, OCH_3_), 3.73–3.78 (m, 1H, H-3), 3.46 (s, 3H, CO_2_CH_3_), 3.37 (dd, *J* = 13.0, 9.0 Hz, 1H, H-2); HRMS *m/z* calcd for C_23_H_22_O_8_NCl_2_ ([M+H]^+^) 510.0717, found 510.0712.

*Data for* **IIb**: Yield = 52%, white solid. m.p. 168–169 °C, [α]^20^_D_ = −69 (*c* 2.7 mg/mL, CHCl_3_); IR cm^−1^ (KBr): 3085, 2935, 1732, 1481, 1228, 1034; ^1^H NMR (500 MHz, CDCl_3_) *δ*: 7.38 (s, 1H, H-5), 6.31 (s, 1H, H-8), 5.97 (dd, *J* = 3.5, 1.0 Hz, 2H, OCH_2_O), 5.72 (d, *J* = 9.0 Hz, 1H, H-1), 4.76–4.80 (m, 1H, H-11), 4.60–4.67 (m, 1H, H-11), 3.95–4.00 (m, 2H, CO_2_C*H*_2_CH_3_), 3.93 (s, 3H, OCH_3_), 3.92 (s, 3H, OCH_3_), 3.80 (s, 3H, OCH_3_), 3.75–3.78 (m, 1H, H-3), 3.34 (dd, *J* = 13.0, 9.0 Hz, 1H, H-2), 1.09 (t, *J* = 7.0 Hz, 3H, CO_2_CH_2_C*H*_3_); HRMS *m/z* calcd for C_24_H_24_O_8_NCl_2_ ([M+H]^+^) 524.0873, found 524.0869.

*Data for* **IIc**: Yield = 79%, white solid, m.p. 97–98 °C, [α]^20^_D_ = −144 (*c* 3.0 mg/mL, CHCl_3_); IR cm^−1^ (KBr): 3078, 2933, 1731, 1479, 1226, 1021; ^1^H NMR (500 MHz, CDCl_3_) *δ*: 7.38 (s, 1H, H-5), 6.32 (s, 1H, H-8), 5.97 (d, *J *= 3.0 Hz, 2H, OCH_2_O), 5.70 (d, *J *= 8.5 Hz, 1H, H-1), 4.75–4.79 (m, 1H, H-11), 4.59–4.66 (m, 1H, H-11), 3.94 (s, 3H, OCH_3_), 3.91 (s, 3H, OCH_3_), 3.78–3.80 (m, 4H, OCH_3_ and H-3), 3.71–3.77 (m, 2H, CO_2_C*H*_2_CH_2_CH_2_CH_3_), 3.38 (dd, *J* = 13.0, 8.0 Hz, 1H, H-2), 1.38–1.47 (m, 2H, CO_2_CH_2_C*H*_2_C*H*_2_CH_3_), 1.23–1.29 (m, 2H, CO_2_CH_2_CH_2_C*H*_2_CH_3_), 0.88 (t, *J *= 7.0 Hz, 3H, CO_2_CH_2_CH_2_CH_2_C*H*_3_); HRMS *m/z* calcd for C_26_H_28_O_8_NCl_2_ ([M+H]^+^) 552.1186, found 552.1176.

*Data for* **IIIa**: Yield = 55%, white solid, m.p. 147–148 °C, [α]^20^_D_ = −85 (c 2.1 mg/mL, CHCl_3_); IR cm^−1^ (KBr): 3045, 2931, 1731, 1483, 1230, 1106; ^1^H NMR (500 MHz, CDCl_3_) *δ*: 7.43 (s, 1H, H-5), 6.52 (s, 1H, H-8), 6.14 (s, 1H, H-6′), 5.99 (dd, *J* = 9.0, 1.0 Hz, 2H, OCH_2_O), 5.38 (d, *J* = 6.0 Hz, 1H, H-1), 4.80–4.83 (m, 1H, H-11), 4.10–4.17 (m, 1H, H-11), 3.88 (s, 3H, OCH_3_), 3.85 (s, 3H, OCH_3_), 3.76–3.81 (m, 1H, H-3), 3.63 (s, 3H, OCH_3_), 3.60 (s, 3H, CO_2_CH_3_), 3.26 (dd, *J *= 12.5, 6.0 Hz, 1H, H-2); HRMS *m/z* calcd for C_23_H_23_O_8_NBr ([M+H]^+^) 520.0602, found 520.0594.

*Data for* **IIIb**: Yield = 49%, white solid, m.p. 148–149 °C, [α]^20^_D_ = −116 (c 3.0 mg/mL, CHCl_3_); IR cm^−1^ (KBr): 3058, 2933, 1726, 1482, 1229, 1106; ^1^H NMR (500 MHz, CDCl_3_) *δ*: 7.42 (s, 1H, H-5), 6.54 (s, 1H, H-8), 6.17 (s, 1H, H-6′), 5.99 (dd, *J* = 10.5, 1.0 Hz, 2H, OCH_2_O), 5.41 (d, *J *= 6.5 Hz, 1H, H-1), 4.81–4.84 (m, 1H, H-11), 4.09–4.17 (m, 2H, CO_2_C*H*_2_CH_3_), 3.92–3.96 (m, 1H, H-11), 3.88 (s, 3H, OCH_3_), 3.85 (s, 3H, OCH_3_), 3.78–3.82 (m, 1H, H-3), 3.64 (s, 3H, OCH_3_), 3.25 (dd, *J* = 12.5, 6.0 Hz, 1H, H-2), 1.18 (t, *J* = 7.0 Hz, 3H, CO_2_CH_2_C*H*_3_); HRMS *m/z* calcd for C_24_H_25_O_8_NBr ([M+H]^+^) 534.0758, found 534.0749.

*Data for* **IIIc**: Yield = 47%, white solid, m.p. 129–130 °C, [α]^20^_D_ = −109 (c 2.3 mg/mL, CHCl_3_); IR cm^−1^ (KBr): 3072, 2938, 1727, 1481, 1226, 1107; ^1^H NMR (500 MHz, CDCl_3_) *δ*: 7.42 (s, 1H, H-5), 6.54 (s, 1H, H-8), 6.16 (s, 1H, H-6′), 5.99 (dd, *J *= 10.0, 1.0 Hz, 2H, OCH_2_O), 5.39 (d, *J* = 6.0 Hz, 1H, H-1), 4.79–4.82 (m, 1H, H-11), 4.11–4.18 (m, 1H, H-11), 4.01–4.05 (m, 1H, CO_2_C*H*_2_CH_2_CH_2_CH_3_), 3.85–3.89 (m, 7H, 2 × OCH_3_, CO_2_C*H*_2_CH_2_CH_2_CH_3_), 3.78–3.82 (m, 1H, H-3), 3.64 (s, 3H, OCH_3_), 3.26 (dd, *J* = 12.5, 6.0 Hz, 1H, H-2), 1.46–1.58 (m, 2H, CO_2_CH_2_C*H*_2_CH_2_CH_3_), 1.26–1.30 (m, 2H, CO_2_CH_2_CH_2_C*H*_2_CH_3_), 0.90 (t, *J *= 7.0 Hz, 3H, CO_2_CH_2_CH_2_CH_2_C*H*_3_); HRMS *m/z* calcd for C_26_H_29_O_8_NBr ([M+H]^+^) 562.1071, found 562.1057.

### General procedure for synthesis of oxime sulfonates of 2′(2′,6′)-(di)halogenopodophyllones (IVa–c; Va–c; and VIb,c)

To a stirred solution of NaH (1.4 mmol) in dry THF (8 mL) at −15 °C was slowly added compound **13**, **14**, or **15** (0.2 mmol). After addition, the reaction mixture was stirred at −15 °C for 0.5 h. Then, the corresponding sulfonyl chlorides (0.8 mmol) were added to the above mixture. After adding, the reaction temperature was raised from −15 °C to room temperature. When the reaction mixture was complete, checked by TLC analysis, saturated aq. NaHCO_3_ (15 mL) was added to the mixture, which was extracted with DCM (3 × 30 mL). The combined organic phase was dried over anhydrous Na_2_SO_4_, filtered, concentrated under reduced pressure, and purified by PTLC to give compounds **IVa–c; Va–c;** and **VIb,c** in 43–72% yields. The example data of **IVa; Va;** and **VIb** are listed as follows, whereas data of **IVb,c; Vb,c;** and **VIc** can be found in the Supporting Information.

*Data for* **IVa**: Yield = 58%, white solid, m.p. 110–111 °C, [α]^20^_D_ = 5.5 (c 2.8 mg/mL, CHCl_3_): IR cm^−1^ (KBr): 3066, 2931, 1778, 1484, 1398, 1194, 1035, 759, 722; ^1^H NMR (500 MHz, CDCl_3_) *δ*: 8.02–8.04 (m, 2H, Ar-H), 7.60–7.63 (m, 3H, Ar-H), 7.14 (s, 1H, H-5), 6.71 (s, 1H, H-8), 6.03 (s, 2H, OCH_2_O), 5.76 (s, 1H, H-6′), 5.07 (d, *J *= 2.0 Hz, 1H, H-1), 4.52–4.55 (m, 1H, H-11), 4.32 (d, *J* = 10.0 Hz, 1H, H-11), 3.92 (s, 3H, OCH_3_), 3.88–3.91 (m, 1H, H-3), 3.84 (s, 3H, OCH_3_), 3.36 (s, 3H, OCH_3_), 3.41 (dd, *J* = 8.5, 2.0 Hz, 1H, H-2); HRMS *m/z* calcd for C_28_H_25_O_10_NClS ([M+H]^+^) 602.0882, found 602.0877.

*Data for* **Va:** Yield = 45%, white solid, m.p. 107–108 °C, [α]^20^_D_ = 26 (c 3.0 mg/mL, CHCl_3_) δ: IR cm^−1^ (KBr): 3067, 2936, 1780, 1481, 1377, 1193, 1021, 722, 686; ^1^H NMR (500 MHz, CDCl_3_) *δ*: 8.05–8.07 (m, 2H, Ar-H), 7.60–7.73 (m, 3H, Ar-H), 7.32 (s, 1H, H-5), 6.20 (s, 1H, H-8), 5.99 (dd, *J* = 10.5, 1.0 Hz, 2H, OCH_2_O), 5.22 (d, *J* = 10.0 Hz, 1H, H-1), 4.95–4.98 (m, 1H, H-11), 4.21–4.26 (m, 1H, H-11), 3.98 (s, 3H, OCH_3_), 3.87–3.94 (m, 7H, 2 × OCH_3_ and H-3), 3.49–3.52 (m, 1H, H-2); HRMS *m/z* calcd for C_28_H_24_O_10_NCl_2_S ([M+H]^+^) 636.0492, found 636.0475.

*Data for* **VIb**: Yield = 44%, yellow solid, m.p. 111–112 °C, [α]^20^_D_ = 22 (2.9 mg/mL, CHCl_3_); IR cm^−1^ (KBr): 3083, 2933, 1781, 1483, 1390, 1194, 1035, 818; ^1^H NMR (500 MHz, CDCl_3_) *δ*: 7.92 (d, *J *= 8.5 Hz, 2H, Ar-H), 7.40 (d, *J* = 8.5 Hz, 2H, Ar-H), 7.15 (s, 1H, H-5), 6.71 (s, 1H, H-8), 6.02 (s, 2H, OCH_2_O), 5.81 (s, 1H, H-6′), 5.08 (d, *J* = 2.5 Hz, 1H, H-1), 4.50–4.54 (m, 1H, H-11), 4.31 (d, *J* = 10.0 Hz, 1H, H-11), 3.91 (s, 3H, OCH_3_), 3.86–3.89 (m, 1H, H-3), 3.84 (s, 3H, OCH_3_), 3.42 (dd, *J *= 8.5, 2.5 Hz, 1H, H-2), 3.36 (s, 3H, OCH_3_), 2.49 (s, 3H, CH_3_); HRMS *m/z* calcd for C_29_H_27_O_10_NBrS ([M+H]^+^) 660.0534, found 660.0516.

### Biological assay

The pesticidal activity of **1;4–12; Ia–c,e–g**; **IIa–f**; **IIIa–g**; **IVa–c**; **Va–c**; **VIb;** and **VIc** was tested as the mortality rate values by using the leaf-dipping method^28^, against the pre-third-instar larvae of *Mythimna separata*. For each compound, 30 pre-third-instar larvae (10 larvae per group) were used. Acetone solutions of **1;4–12; Ia–c,e–g**; **IIa–f**; **IIIa–g**; **IVa–c**; **Va–c**; **VIb; VIc;** and toosendanin (a positive control) were prepared at 1 mg/mL. Fresh wheat leaf discs (1 × 1 cm) were dipped into the corresponding solution for 3 s, then taken out and dried. Leaf discs treated with acetone alone were used as a blank control group. Several pieces of treated leaf discs were kept in each dish (10 larvae per dish), which was then placed in a conditioned room (25 ± 2 °C, 65–80% relative humidity, 12 h/12 h (light/dark) photoperiod). If the treated leaf discs were consumed, additional treated ones were added to the dish. After 48 h, untreated fresh leaves were added to all dishes till the end of pupae. The corrected mortality rate values were obtained by the formula:


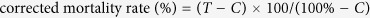


where *T* is the mortality rate in the group treated with the tested compounds, and *C* is the mortality rate in the blank control group (*T* and *C* were all expressed as the percentage).

## Results and Discussion

**Figure 2:**
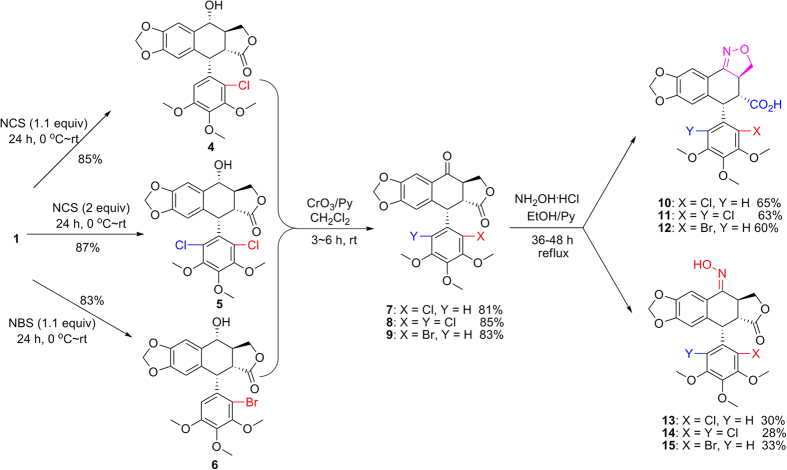
The synthetic route for the preparation of compounds 10–15.

**Figure 3:**
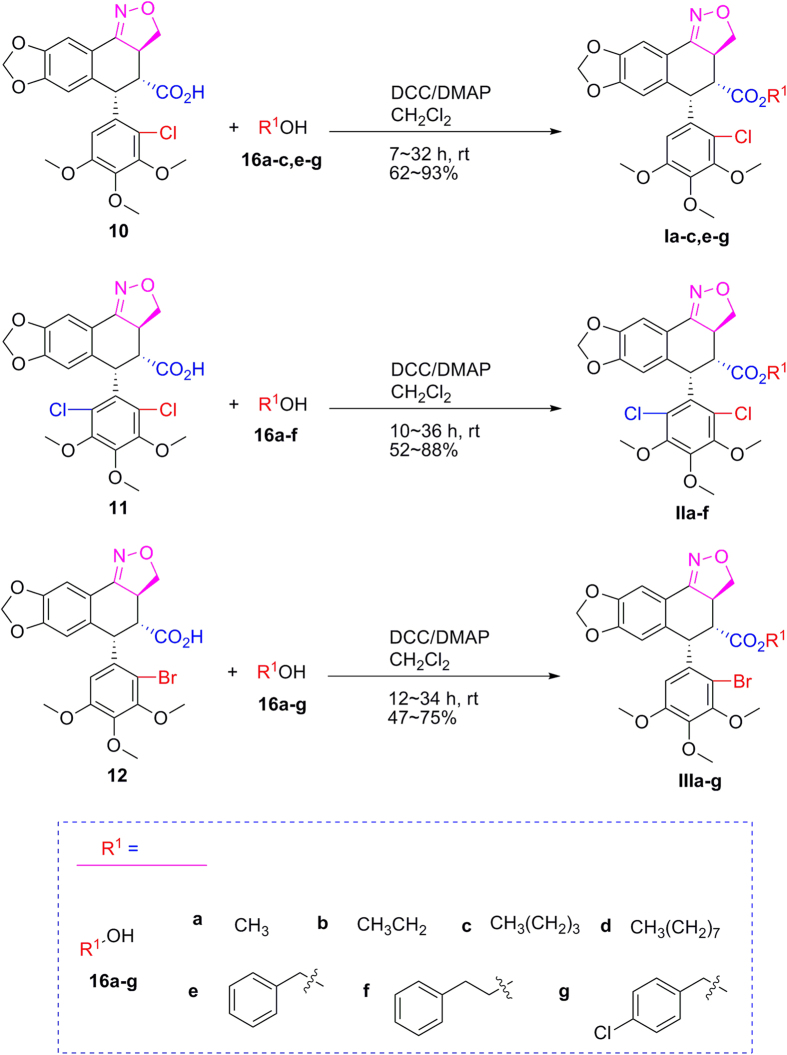
Preparation of 2′(2′,6′)-(di)halogeno-isoxazolopodophyllic acid-based esters (I–III).

**Figure 4:**
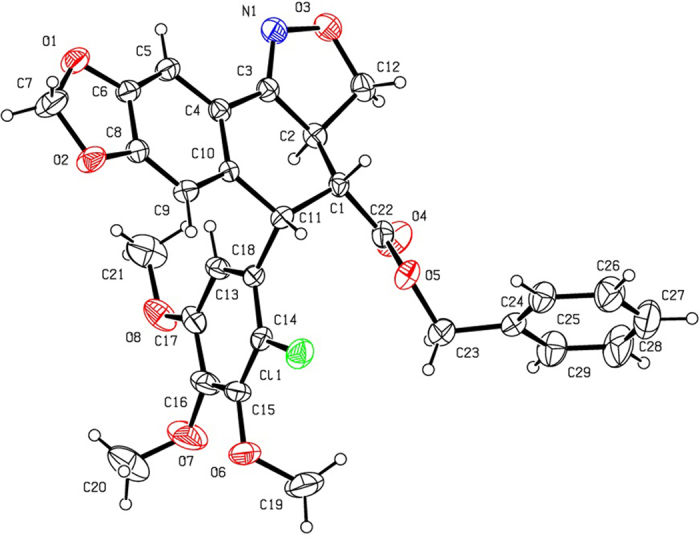
The X-ray crystal structure of Ie. Drawing by Hui Xu.

**Figure 5:**
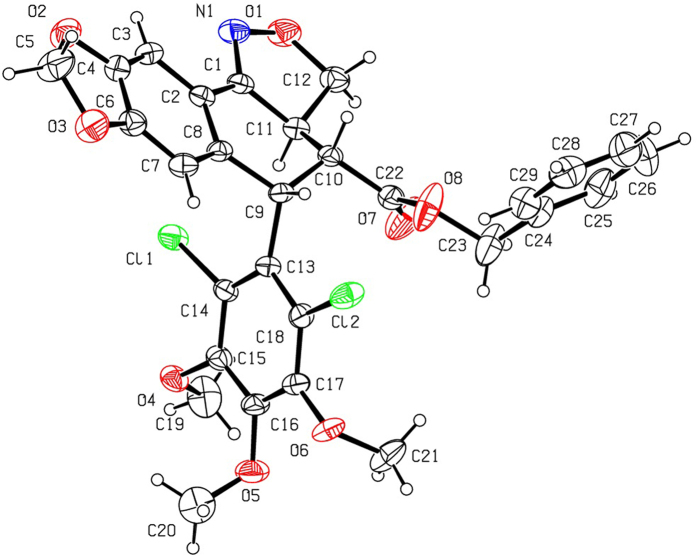
The X-ray crystal structure of IIe. Drawing by Hui Xu.

**Figure 6:**
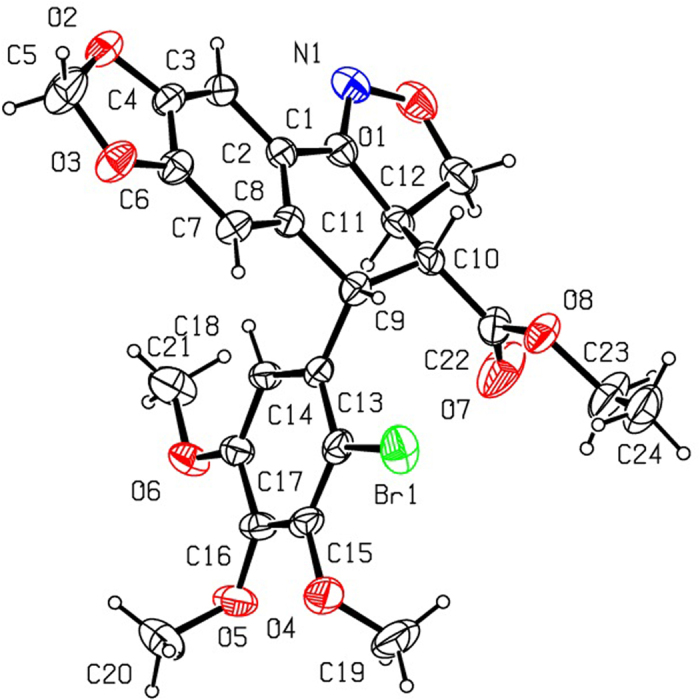
The X-ray crystal structure of IIIb. Drawing by Hui Xu.

As shown in Fig. 2, 2′-chloropodophyllotoxin (**4**), 2′,6′-dichloropodophyllotoxin (**5**), and 2′-bromopodophyllotoxin (**6**) were firstly obtained as described previously^28^. Then, 2′(2′,6′)-(di)halogenopodophyllones (**7–9**) were easily obtained by oxidation of **4**–**6**, respectively. Subsequently, when compounds **7**–**9** reacted with hydroxylamine hydrochloride, 2′(2′,6′)-(di)halogeno-isoxazolopodophyllic acids (**10**–**12**) and oximes of 2′(2′,6′)-(di)halogenopodophyllones (**13**–**15**) were all produced. However, in our previous paper, when 2′(2′,6′)-(di)chloropicropodophyllones reacted with hydroxylamine hydrochloride, only oximes of 2′(2′,6′)-(di)chloropicropodophyllotoxin were afforded^24^. It indicated that when 2′(2′,6′)-(di)halogenopodophyllones or 2′(2′,6′)-(di)chloropicropodophyllones reacted with hydroxylamine hydrochloride, the desired products were related with the configuration of their lactones. Finally, as shown in Fig. 3, 2′(2′,6′)-(di)halogeno-isoxazolopodophyllic acids-based esters (**Ia–c,e–g**; **IIa–f**; and **IIIa–g**) were prepared by the reaction of **10**–**12** with different alcohols in the presence of DCC and DMAP, and well characterized by ^1^H NMR, HRMS, optical rotation, mp and IR. Especially three single-crystal structures of **Ie**, **IIe** and **IIIb** were determined by X-ray crystallography as illustrated in Figs 4, 5, 6, respectively. It showed that the chlorine atom of **Ie** was at the C-2′ position; two chlorine atoms of **IIe** was at the C-2′ and C-6′ position; the bromine atom of **IIIb** was at the C-2′ position. Meanwhile, the two hydrogen atoms at C-2 and C-3 position of **Ie**, **IIe** and **IIIb** were all in β and α configuration, respectively. Crystallographic data (excluding structure factors) for the structures of **Ie**, **IIe** and **IIIb** have been deposited at the Cambridge Crystallographic Data Centre with supplementary publication number CCDC 1482635, 1482788, and 1482789, respectively. Copies of the data can be obtained, free of charge, on application to CCDC, 12 Union Road, Cambridge CB2 1EZ, UK [fax: +44 (0)1223 336033 or e-mail: deposit@ccdc.cam.ac.uk].

**Figure 7:**
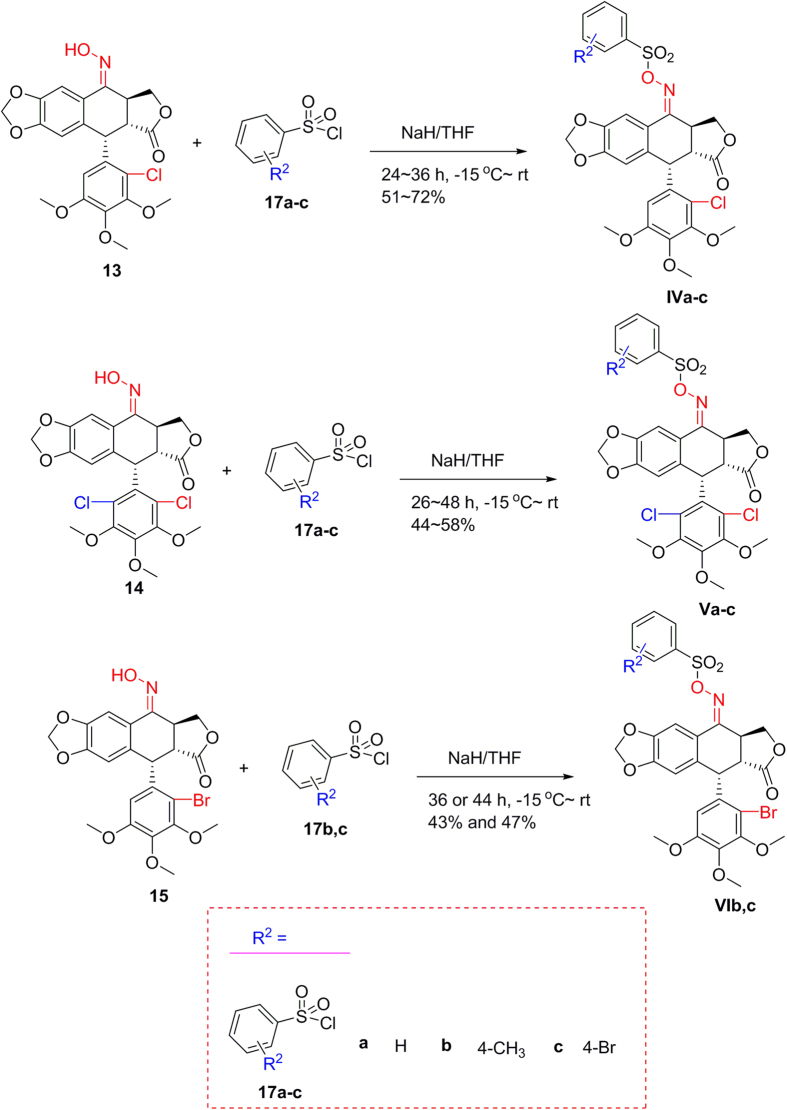
Preparation of oxime sulfonate derivatives of 2′(2′,6′)-(di)chloropodophyllotoxins (IV–VI).

On the other hand, as described in Fig. 7, oxime sulfonates of 2′(2′,6′)-(di)halogenopodophyllones (**IVa–c**; **Va–c**; and **VIb,c**) were smoothly obtained by reaction of **13**–**15** with the corresponding sulfonyl chlorides. Their structures were well characterized by ^1^H NMR, HRMS, optical rotation, mp and IR.

**Figure 8:**
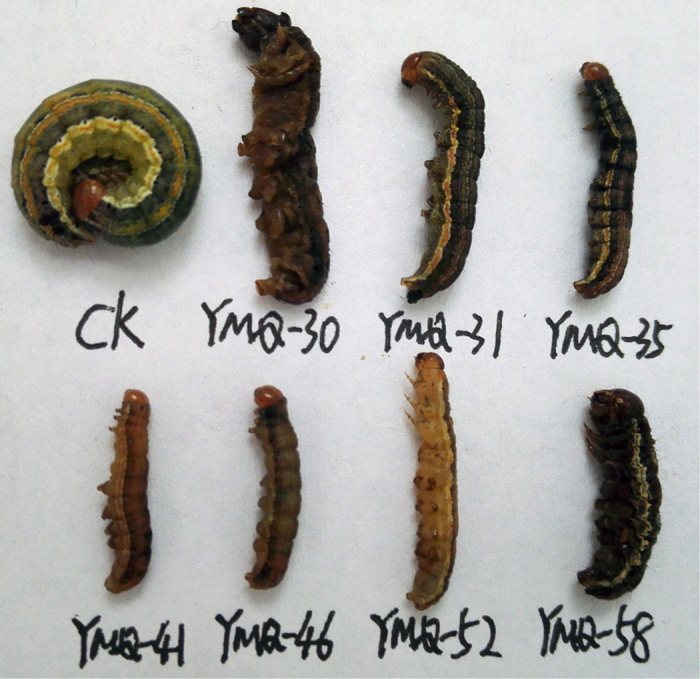
Representative abnormal larvae pictures of compounds Ic (YMQ-30), If (YMQ-31), IVb (YMQ-35), IIc (YMQ-41), IIIa (YMQ-46), IIIg (YMQ-52), and Va (YMQ-58) during the larval period (CK = blank control group).

**Figure 9:**
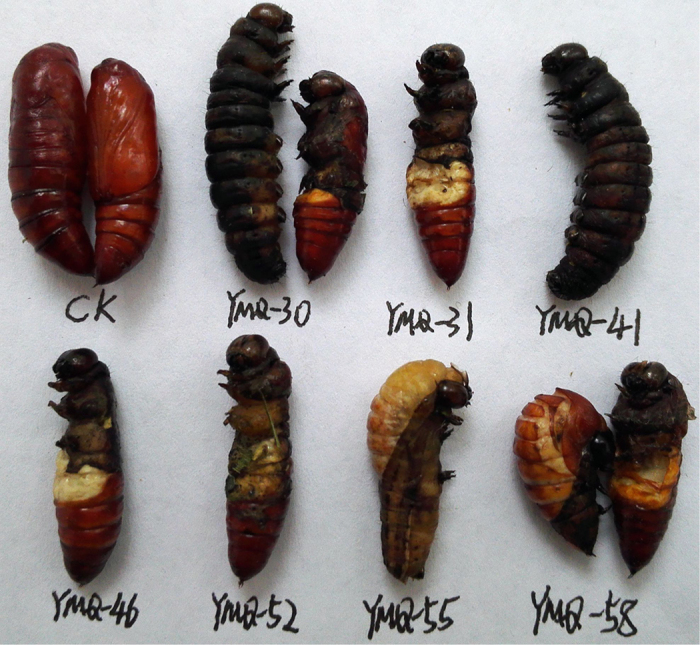
Representative malformed pupae pictures of compounds Ic (YMQ-30), If (YMQ-31), IIc (YMQ-41), IIIa (YMQ-46), IIIg (YMQ-52), Vb (YMQ-55), and Va (YMQ-58) during the pupation period (CK = blank control group).

**Figure 10:**
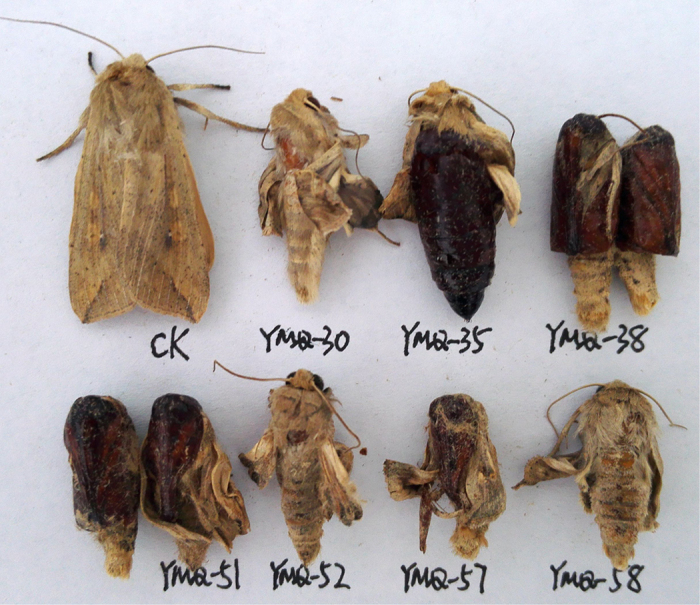
Representative malformed moth pictures of compounds Ic (YMQ-30), IVb (YMQ-35), IIb (YMQ-38), IIIf (YMQ-51), IIIg (YMQ-52), IId (YMQ-57), and Va (YMQ-58) during the stage of adult emergence (CK = blank control group).

As shown in Table 1, the insecticidal activity of **1;4–12; Ia–c,e–g; IIa–f; IIIa–g; IVa–c; Va–c; VIb;** and **VIc** against the pre-third-instar larvae of *M. separata in vivo* was evaluated at a concentration of 1 mg/mL. The corresponding mortality rates after 35 days were higher than those after 10 and 20 days. For example, the mortality rate of compound **IIc** against *M. separata* after 35 days was 62.0%, whereas the mortality rates of **IIc** against *M. separata* after 10 and 20 days were 16.7% and 36.7%, respectively. It suggested that these podophyllotoxin compounds showed delayed insecticidal activity^24^,^28^. Meanwhile, the symptoms of the treated *M. separata* were observed in the same way as our previous reports^24^,^28^. As shown in Fig. 8, many larvae of the treated groups died slowly during the larval stage; as shown in Fig. 9, some malformed pupae of the treated groups also appeared and died during the pupation stage; some malformed moths with imperfect wings also appeared in the treated groups (Fig. 10).

Among all derivatives, compounds **4; 5; 7; 9; 11; Ia; Ic; If; IIa–f; IIIb; IIIc; IIIe; IIIf; IVb; IVc; Va–c; VIb;** and **VIc** exhibited equal or higher insecticidal activity than toosendanin. Compounds **IIc** and **Vc**, especially, showed the highest insecticidal activity. For example, the final mortality rates (FMRs) of **IIc** and **Vc** were 62.0%, and 65.2%, respectively. Introduction of a halogen atom on the E ring of podophyllotoxin/podophyllotoxone was important for the insecticidal activity. For example, FMRs of **4** (containing a 2′-chlorine atom), **5** (containing two 2′,6′-dichlorine atoms) and **6** (containing a 2′-bromine atom) were 44.8%, 51.7%, and 41.4%, respectively; whereas the FMR of **1** was only 34.5%. FMRs of **7** (containing a 2′-chlorine atom), **8** (containing two 2′,6′-dichlorine atoms) and **9** (containing a 2′-bromine atom) were 44.8%, 51.7%, and 41.4%, respectively; whereas the FMR of podophyllotoxone was only 17.2%^29^. In general, 2′,6′-dichloro-isoxazolopodophyllic acids-based esters exhibited more potent insecticidal activities than those of the corresponding 2′-chloro/bromo-isoxazolopodophyllic acids-based ones. For example, FMRs of **IIa–c,e,f** were 55.2%, 55.2%, 62.0%, 44.8% and 55.2%, respectively; whereas FMRs of **Ia–c,e,f** were 44.8%, 37.9%, 44.8%, 37.9% and 44.8%, respectively. To oxime sulfonates of 2′(2′,6′)-(di)halogenopodophyllones (**IVa–c**; **Va–c**; and **VIb,c**), introduction of a bromine atom on the phenyl ring of the sulfonate moiety led to more potent compounds than those containing methyl or hydrogen ones (**IVc** vs **IVa** and **IVb**; **Vc** vs **Va** and **Vb**; **VIc** vs **VIb**).

## Conclusion

In summary, a series of 2′(2′,6′)-(di)halogeno-isoxazolopodophyllic acids-based esters and oxime sulfonates of 2′(2′,6′)-(di)halogenopodophyllones were prepared, and evaluated for their insecticidal activity against the pre-third-instar larvae of *M. separata in vivo*. It suggested that when 2′(2′,6′)-(di)halogenopodophyllones or 2′(2′,6′)-(di)chloropicropodophyllones reacted with hydroxylamine hydrochloride, the desired products were determined by the configuration of their lactones. Three key single-crystal structures of **Ie**, **IIe** and **IIIb** were confirmed by X-ray crystallography.

Among all derivatives, especially compounds **IIc** and **Vc** showed the highest insecticidal activity. Moreover, some interesting results of structure-insecticidal activity relationships of tested compounds were also observed. This will pave the way for further design and structural modifications of podophyllotoxin derivatives as insecticidal agents.

## Additional Information

**How to cite this article**: Yu, M. *et al*. Design, Synthesis and Evaluation of Novel Isoxazolines/Oxime Sulfonates of 2′(2′,6′)-(Di)Chloropodophyllotoxins as Insecticidal Agents. *Sci. Rep.* **6**, 33062; doi: 10.1038/srep33062 (2016).

## Supplementary Material

Supplementary Information

## Figures and Tables

**Figure 1:**
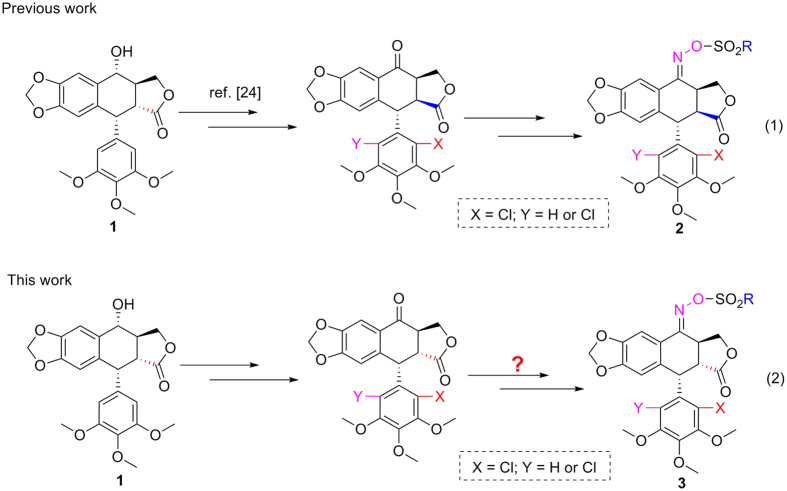
Chemical structures of podophyllotoxin (1) and its derivatives (2 and 3).

**Table 1:** Insecticidal Activity of 1; 4–12; Ia–c,e–g; IIa–f; IIIa–g; IVa–c; Va–c; VIb; and VIc against *M. separata* on Leaves Treated with a Concentration of 1 mg/mL.

Compound	Corrected mortality rate (%)		
	10 days	20 days	35 days
**1**	3.3 ± 3.3	23.3 ± 3.3	34.5 ± 3.3
**4**	23.3 ± 6.7	26.7 ± 8.8	44.8 ± 3.3
**5**	16.7 ± 3.3	36.7 ± 3.3	51.7 ± 3.3
**6**	26.7 ± 8.8	36.7 ± 3.3	41.4 ± 3.3
**7**	10.0 ± 5.8	33.3 ± 6.7	48.2 ± 5.8
**8**	13.3 ± 3.3	30.0 ± 5.8	41.4 ± 8.8
**9**	10.0 ± 5.8	33.3 ± 3.3	44.8 ± 3.3
**10**	13.3 ± 8.8	30.0 ± 5.8	41.4 ± 3.3
**11**	26.7 ± 3.3	33.3 ± 6.7	51.7 ± 3.3
**12**	16.7 ± 3.3	23.3 ± 6.7	37.9 ± 5.8
**Ia**	16.7 ± 3.3	20.0 ± 0	44.8 ± 3.3
**Ib**	10.0 ± 5.8	20.0 ± 5.8	37.9 ± 5.8
**Ic**	26.7 ± 3.3	36.7 ± 6.7	44.8 ± 3.3
**Ie**	10.0 ± 5.8	26.7 ± 3.3	37.9 ± 5.8
**If**	23.3 ± 3.3	30.0 ± 5.8	44.8 ± 3.3
**Ig**	20.0 ± 0	26.7 ± 6.7	31.0 ± 8.8
**IIa**	20.0 ± 0	30.0 ± 0	55.2 ± 3.3
**IIb**	16.7 ± 3.3	36.7 ± 8.8	55.2 ± 6.7
**IIc**	16.7 ± 3.3	36.7 ± 3.3	62.0 ± 3.3
**IId**	10.0 ± 0	33.3 ± 6.7	48.2 ± 5.8
**IIe**	6.7 ± 3.3	30.0 ± 5.8	44.8 ± 3.3
**IIf**	16.7 ± 3.3	20.0 ± 0	55.2 ± 3.3
**IIIa**	20.0 ± 5.8	33.3 ± 8.8	41.4 ± 6.7
**IIIb**	13.3 ± 3.3	20.0 ± 0	48.2 ± 3.3
**IIIc**	20.0 ± 0	26.7 ± 3.3	51.7 ± 3.3
**IIId**	13.3 ± 3.3	23.3 ± 3.3	41.4 ± 3.3
**IIIe**	6.7 ± 3.3	26.7 ± 6.7	51.7 ± 3.3
**IIIf**	23.3 ± 3.3	36.7 ± 6.7	44.8 ± 8.8
**IIIg**	16.7 ± 3.3	26.7 ± 6.7	37.9 ± 5.8
**IVa**	13.3 ± 3.3	23.3 ± 3.3	34.5 ± 3.3
**IVb**	16.7 ± 3.3	33.3 ± 6.7	51.7 ± 3.3
**IVc**	20.0 ± 5.8	26.7 ± 3.3	58.6 ± 0
**Va**	16.7 ± 3.3	40.0 ± 5.8	44.8 ± 6.7
**Vb**	23.3 ± 8.8	33.3 ± 3.3	44.8 ± 3.3
**Vc**	16.7 ± 3.3	30.0 ± 5.8	65.2 ± 6.7
**VIb**	10.0 ± 0	20.0 ± 5.8	44.8 ± 3.3
**VIc**	23.3 ± 8.8	36.7 ± 3.3	55.2 ± 3.3
toosendanin	16.7 ± 3.3	33.3 ± 3.3	44.8 ± 3.3
