# Supplementary material for: Design, Synthesis and Evaluation of Novel Isoxazolines/Oxime Sulfonates of 2′(2′,6′)-(Di)Chloropodophyllotoxins as Insecticidal Agents
Source: Sci Rep. 2016 Sep 26;6:33062. doi: 10.1038/srep33062 (PMC5036094; doi:10.1038/srep33062)
Supplement: Supplementary Information [file srep33062-s1.doc]

Design, Synthesis and Evaluation of Novel Isoxazolines/Oxime Sulfonates of 2′(2′,6′)-(Di)Chloropodophyllotoxins as Insecticidal Agents

Mingqiao Yu^1^, Guangci Liu^1^, Yuanyuan Zhang^1^, Tao Feng^1^, Ming Xu^1^, and Hui Xu^1,2^

^1^Research Institute of Pesticidal Design & Synthesis, College of Sciences, Northwest A&F University, Yangling 712100, Shaanxi Province, P. R. China.

^2^Shaanxi Key Laboratory of Natural Products & Chemical Biology, College of Plant Protection, Northwest A&F University, Yangling 712100, Shaanxi Province, P. R. China.

Correspondence and requests for materials should be addressed to H.X. ([orgxuhui@nwsuaf.edu.cn](mailto:orgxuhui@nwsuaf.edu.cn)); Telephone: +86(0)29-87091952; Fax: +86(0)29-87091952.

***Data for Ie:*** Yield = 93%, white solid, m.p. 169-170 °C, [α]^20^_D_ = -90 (c 3.0 mg/mL, CHCl_3_); IR cm^-1^ (KBr): 3076, 2984, 2932, 1728, 1623, 1557, 1451, 1392, 1183, 1108, 738, 699, 662; ^1^H NMR (500 MHz, CDCl_3_) δ: 7.43 (s, 1H, H-5), 7.32-7.47 (m, 3H, Ar-H), 7.25-7.26 (m, 2H, Ar-H), 6.50 (s, 1H, H-8), 6.09 (s, 1H, H-6’), 5.98 (d, *J* = 6.5 Hz, 2H, OCH_2_O), 5.34 (d, *J* = 5.5 Hz, 1H, H-1), 5.13 (d, *J* = 12.0 Hz, 1H, C*H*_2_Ph), 4.90 (d, *J* = 12.0 Hz, 1H, C*H*_2_Ph), 4.73-4.77 (m, 1H, H-11), 4.08-4.15 (m, 1H, H-11), 3.88 (s, 3H, OCH_3_), 3.85 (s, 3H, OCH_3_), 3.76-3.80 (m, 1H, H-3), 3.62 (s, 3H, OCH_3_), 3.28-3.32 (m, 1H, H-2); HRMS *m/z* calcd for C_29_H_27_O_8_NCl ([M+H]^+^) 552.1420, found 552.1411.

***Data for If:*** Yield = 86%, pale yellow syrup liquid, [α]^20^D = -91 (c 3.0 mg/mL, CHCl_3_); IR cm-1 (KBr): 3061, 3026, 2938, 1734, 1621, 1569, 1482, 1395, 1231, 1109, 749, 701, 612; ^1^H NMR (500 MHz, CDCl_3_) δ: 7.42 (s, 1H, H-5), 7.26-7.32 (m, 3H, Ar-H), 7.14 (d, *J* = 6.5 Hz, 2H, Ar-H), 6.50 (s, 1H, H-8), 6.07 (s, 1H, H-6’), 5.99 (dd, *J* = 8.0, 1.0 Hz, 2H, OCH_2_O), 5.30 (d, *J* = 5.5 Hz, 1H, H-1), 4.59-4.63 (m, 1H, H-11), 4.27-4.32 (m, 1H, H-11), 4.02-4.09 (m, 2H, CO_2_C*H*_2_CH_2_Ph), 3.88 (s, 3H, OCH_3_), 3.84 (s, 3H, OCH_3_), 3.65-3.69 (m, 1H, H-3), 3.62 (s, 3H, OCH_3_), 3.18-3.22 (m, 1H, H-2), 2.89 (t, *J* = 6.5 Hz, 2H, CO_2_CH_2_C*H*_2_Ph); HRMS m/z calcd for C_30_H_29_O_8_NCl ([M+H]+) 566.1576, found 566.1563.

***Data for Ig:*** Yield = 62%, pale yellow syrup liquid, [α]^20^_D_ = -66 (c 3.0 mg/mL, CHCl_3_); IR cm^-1^ (KBr): 3072, 2934, 1866, 1733, 1623, 1569, 1484, 1397, 1233, 1011, 800, 611; ^1^H NMR (500 MHz, CDCl_3_) δ: 7.42 (s, 1H, H-5), 7.34 (d, *J* = 7.5 Hz, 2H, Ar-H), 7.21 (d, *J* = 8.5 Hz, 2H, Ar-H), 6.50 (s, 1H, H-8), 6.08 (s, 1H, H-6’), 5.99 (dd, *J* = 8.0, 1.0 Hz, 2H, OCH_2_O), 5.33 (d, *J* = 5.5 Hz, 1H, H-1), 5.08 (d, *J* = 12.0 Hz, 1H, C*H*_2_Ph), 4.87 (d, *J* = 12.5 Hz, 1H, C*H*_2_Ph), 4.74-4.77 (m, 1H, H-11), 4.07-4.14 (m, 1H, H-11), 3.88 (s, 3H, OCH_3_), 3.85 (s, 3H, OCH_3_), 3.75-3.79 (m, 1H, H-3), 3.62 (s, 3H, OCH_3_), 3.27-3.31 (m, 1H, H-2); HRMS *m/z* calcd for C_29_H_26_O_8_NCl_2_ ([M+H]^+^) 586.1030, found 586.1019.

***Data for IId:*** Yield = 53%, pale yellow syrup liquid, [α]^20^_D_ = -72 (c 2.7 mg/mL, CHCl_3_); IR cm^-1^ (KBr): 3094, 2930, 1857, 1732, 1621, 1556, 1478, 1302, 1228, 1025, 778; ^1^H NMR (500 MHz, CDCl_3_) δ: 7.38 (s, 1H, H-5), 6.32 (s, 1H, H-8), 5.98 (dd, *J* = 4.0, 1.0 Hz, 2H, OCH_2_O), 5.70 (d, *J* = 9.0 Hz, 1H, H-1), 4.75-4.79 (m, 1H, H-11), 4.59-4.66 (m, 1H, H-11), 3.94 (s, 3H, OCH_3_), 3.91 (s, 3H, OCH_3_), 3.80 (s, 3H, OCH_3_), 3.76-3.78 (m, 1H, H-3), 3.63-3.72 (m, 2H, CO_2_C*H*_2_C_7_H_15_), 3.37 (dd, *J* = 13.0, 9.0 Hz, 1H, H-2), 1.54-1.58 (m, 2H, CO_2_CH_2_C*H*_2_(CH_2_)_5_CH_3_), 1.28-1.44 (m, 10H, CO_2_CH_2_CH_2_(C*H*_2_)_5_CH_3_), 0.89 (t, *J* = 7.0 Hz, 3H, CO_2_CH_2_CH_2_(CH_2_)_5_C*H*_3_); HRMS *m/z* calcd for C_30_H_36_O_8_NCl_2_ ([M+H]^+^) 608.1812, found 608.1794.

***Data for IIe:*** Yield = 88%, pale yellow solid, m.p. 135-137 °C, [α]^20^_D_ = -115 (c 2.8 mg/mL, CHCl_3_); IR cm^-1^ (KBr): 3064, 2937, 2870, 1733, 1623, 1558, 1479, 1396, 1229, 1019, 740, 698, 595; ^1^H NMR (500 MHz, CDCl_3_) δ: 7.30-7.36 (m, 4H, H-5 and Ar-H), 7.21-7.23 (m, 2H, Ar-H), 6.32 (s, 1H, H-8), 5.97 (dd, *J* = 4.5, 1.0 Hz, 2H, OCH_2_O), 5.71 (d, *J* = 8.5 Hz, 1H, H-1), 5.00 (d, *J* =7.0 Hz, 1H, H-11), 4.70-4.74 (m, 2H, C*H*_2_Ph), 4.58-4.65 (m, 1H, H-11), 3.93 (s, 3H, OCH_3_), 3.87 (s, 3H, OCH_3_), 3.73-3.76 (m, 4H, H-3 and OCH_3_), 3.38-3.42 (m, 1H, H-2); HRMS *m/z* calcd for C_29_H_26_O_8_NCl_2_ ([M+H]^+^) 586.1030, found 586.1024.

***Data for IIf:*** Yield = 58%, pale yellow syrup liquid, [α]^20^_D_ = -173 (c 3.0 mg/mL, CHCl_3_); IR cm^-1^ (KBr): 3062, 2938, 2868, 1732, 1623, 1550, 1453, 1394, 1227, 1019, 773, 747, 699; ^1^H NMR (500 MHz, CDCl_3_) δ: 7.37 (s, 1H, H-5), 7.26-7.32 (m, 3H, Ar- H), 7.14 (d, *J* = 7.0 Hz, 2H, Ar-H), 6.32 (s, 1H, H-8), 5.97 (dd, *J* = 4.0, 1.0 Hz, 2H, OCH_2_O), 5.69 (d, *J* = 9.0 Hz , 1H, H-1), 4.55-4.61 (m, 1H, H-11), 4.13-4.16 (m, 1H, H-11), 3.92 (s, 3H, OCH_3_), 3.91 (3, 3H, OCH_3_), 3.85-3.88 (m, 2H, CO_2_C*H*_2_CH_2_Ph), 3.80 (s, 3H, OCH_3_), 3.63-3.67 (m, 1H, H-3), 3.34 (dd, *J* = 12.5, 8.5 Hz, 1H, H-2), 2.89 (t, *J* = 7.0 Hz, 2H, CO_2_CH_2_C*H*_2_Ph); HRMS *m/z* calcd for C_30_H_28_O_8_NCl_2_ ([M+H]^+^) 600.1186, found 600.1176.

***Data for IIId:*** Yield = 60%, pale yellow syrup liquid, [α]^20^_D_ = -76 (c 3.0 mg/mL, CHCl_3_); IR cm^-1^ (KBr): 3091, 2925, 2853, 1724, 1622, 1563, 1482, 1394, 1224, 1108, 602; ^1^H NMR (500 MHz, CDCl_3_) δ: 7.42 (s, 1H, H-5), 6.54 (s, 1H, H-8), 6.15 (s, 1H, H-6’), 5.99 (dd, *J* = 10.0, 1.0 Hz, 2H, OCH_2_O), 5.39 (d, *J* = 5.5 Hz, 1H, H-1), 4.79-4.82 (m, 1H, H-11), 4.13-4.18 (m, 1H, H-11), 4.00-4.05 (m, 1H, CO_2_C*H*_2_C_7_H_15_), 3.84-3.88 (m, 7H, CO_2_C*H*_2_C_7_H_15_ and 2×OCH_3_), 3.78-3.82 (m, 1H, H-3), 3.64 (s, 3H, OCH_3_), 3.26 (dd, *J* = 13.0, 6.0 Hz, 1H, H-2), 1.54-1.58 (m, 2H, CO_2_CH_2_C*H*_2_(CH_2_)_5_CH_3_), 1.28-1.36 (m, 10H, CO_2_CH_2_CH_2_(C*H*_2_)_5_CH_3_), 0.89 (t, *J* = 7.0 Hz, 3H, CO_2_CH_2_CH_2_(CH_2_)_5_C*H*_3_); HRMS *m/z* calcd for C_30_H_37_O_8_NBr ([M+H]^+^) 618.1697, found 618.1680.

***Data for IIIe:*** Yield = 59%, pale yellow syrup liquid, [α]^20^_D_ = -73 (c 3.2 mg/mL, CHCl_3_); IR cm^-1^ (KBr): 3063, 2931, 2863, 1731, 1628, 1563, 1481, 1390, 1231, 1104, 737, 697, 592; ^1^H NMR (500 MHz, CDCl_3_) δ: 7.42 (s, 1H, H-5), 7.29-7.36 (m, 5H, Ar-H), 6.52 (s, 1H, H-8), 6.14 (s, 1H, H-6’), 5.98 (dd, *J* = 8.5, 1.0 Hz, 2H, OCH_2_O), 5.41 (d, *J* = 6.0 Hz, 1H, H-1), 5.13 (d, *J* = 12. 0 Hz, 1H, C*H*_2_Ph), 4.89 (d, *J* = 12.0 Hz, 1H, C*H*_2_Ph), 4.74-4.77 (m, 1H, H-11), 4.12-4.19 (m, 1H, H-11), 3.86 (s, 3H, OCH_3_), 3.85 (s, 3H, OCH_3_), 3.75-3.80 (m, 1H, H-3), 3.62 (s, 3H, OCH_3_), 3.31 (dd, *J* = 13.0, 6.0 Hz, 1H, H-2); HRMS *m/z* calcd for C_29_H_27_O_8_NBr ([M+H]^+^) 596.0915, found 596.0902.

***Data for IIIf:*** Yield = 75%, pale yellow syrup liquid, [α]^20^_D_ = -58 (c 3.0 mg/mL, CHCl_3_); IR cm^-1^ (KBr): 3062, 2936, 2872, 1732, 1586, 1564, 1481, 1391, 1232, 1040, 746, 699, 522; ^1^H NMR (500 MHz, CDCl_3_) δ: 7.41 (s, 1H, H-5), 7.25-7.31 (m, 3H, Ar-H), 7.14 (d, *J* = 7.0 Hz, 2H, Ar-H), 6.52 (s, 1H, H-8), 6.14 (s, 1H, H-6’) , 5.99（dd, *J* = 9.5, 1.0 Hz, 2H, OCH_2_O), 5.36 (d, *J* = 5.5 Hz, 1H, H-1), 4.59-4.63 (m, 1H, H-11), 4.26-4.31 (m, 1H, H-11), 4.03-4.08 (m, 2H, CO_2_C*H*_2_CH_2_Ph), 3.86 (s, 3H, OCH_3_), 3.84 (s, 3H, OCH_3_), 3.65-3.69 (m, 1H, H-3), 3.63 (s, 3H, OCH_3_), 3.21 (dd, *J* = 12.5, 6.0 Hz, 1H, H-2), 2.77-2.81 (m, 2H, CO_2_CH_2_C*H*_2_Ph); HRMS *m/z* calcd for C_30_H_29_O_8_NBr ([M+H]^+^) 610.1071, found 610.1057.

***Data for IIIg:*** Yield = 64%, pale yellow syrup liquid, [α]^20^_D_ = -74 (c 3.0 mg/mL, CHCl_3_); IR cm^-1^ (KBr): 3083, 2932, 2857, 1734, 1623, 1564, 1483, 1391, 1232, 1009, 802, 576; ^1^H NMR (500 MHz, CDCl_3_) δ: 7.42 (s, 1H, H-5), 7.31 (d, *J* = 7.0 Hz, 2H, Ar-H), 7.20 (d, *J* = 8.5 Hz, 2H, Ar-H), 6.52 (s, 1H, H-8), 6.14 (s, 1H, H-6’), 5.99 (dd, *J* = 9.0, 1.0 Hz, 2H, OCH_2_O), 5.40 (d, *J* = 6.0 Hz, 1H, H-1), 5.08 (d, *J* = 12.5 Hz, 1H, C*H*_2_Ph), 4.86 (d, *J* = 12.0 Hz, 1H, C*H*_2_Ph), 4.74-4.78 (m, 1H, H-11), 4.11-4.18 (m, 1H, H-11), 3.87 (s, 3H, OCH_3_), 3.85 (s, 3H, OCH_3_), 3.75-3.79 (m, 1H, H-3), 3.63 (s, 3H, OCH_3_), 3.30 (dd, *J* = 13.0, 6.0 Hz, 1H, H-2); HRMS *m/z* calcd for C_29_H_26_O_8_NBrCl ([M+H]^+^) 630.0525, found 630.0508.

***Data for IVb:*** Yield = 52%, yellow solid, m.p. 117-119 °C , [α]^20^_D_ =10 (c 2.4 mg/mL, CHCl_3_); IR cm^-1^ (KBr): 3098, 2935, 2884, 1779, 1733, 1619, 1594, 1484, 1398, 1193, 1036, 819, 664; ^1^H NMR (500 MHz, CDCl_3_) δ: 7.91 (d, *J* = 8.0 Hz, 2H, Ar-H), 7.40 (d, *J* = 8.5 Hz, 2H, Ar-H), 7.15 (s, 1H, H-5), 6.71 (s, 1H, H-8), 6.03 (s, 2H, OCH_2_O), 5.77 (s, 1H, H-6’), 5.07 (d, *J* = 2.5 Hz, 1H, H-1), 4.50-4.54 (m, 1H, H-11), 4.30 (d, *J* = 5.0, 1H, H-11), 3.92 (s, 3H, OCH_3_), 3.87-3.90 (m, 1H, H-3), 3.84 (s, 3H, OCH_3_), 3.37-3.99 (m, 1H, H-2), 3.36 (s, 3H, OCH_3_), 2.49 (s, 3H, CH_3_); HRMS *m/z* calcd for C_29_H_27_O_10_NClS ([M+H]^+^) 616.1039, found 616.1025.

***Data for IVc:*** Yield = 73%, yellow solid , m.p. 118-119 °C , [α]^20^_D_ = 2 (c 3.0 mg/mL, CHCl_3_); IR cm^-1^ (KBr): 3092, 2929, 2891, 1779, 1620, 1575, 1483, 1392, 1193, 1068, 816, 603; ^1^H NMR (500 MHz, CDCl_3_) δ: 7.89 (d, *J* = 8.5 Hz, 2H, Ar-H), 7.76 (d, *J* = 8.5 Hz, 2H, Ar-H), 7.14 (s, 1H, H-5), 6.72 (s, 1H, H-8), 6.03 (s, 2H, OCH_2_O), 5.75 (s, 1H, H-6’), 5.08 (d, *J* = 2.5 Hz, 1H, H-1), 4.51-4.55 (m, 1H, H-11), 4.33 (d, *J* = 10.0 Hz, 1H, H-11), 3.92 (s, 3H, OCH_3_), 3.87-3.90 (m, 1H, H-3), 3.84 (s, 3H, OCH_3_), 3.39-3.41 (m, 1H, H-2), 3.37 (s, 3H, OCH_3_); HRMS *m/z* calcd for C_28_H_24_O_10_NBrClS ([M+H]^+^) 679.999, found 679.997.

***Data for Vb:*** Yield = 49%, yellow solid, m.p. 131-132 °C, [α]^20^_D_ = 17 (c 2.0 mg/mL, CHCl_3_); IR cm^-1^ (KBr): 3085, 2935, 2876, 1780, 1619, 1567, 1482, 1389, 1193, 1105, 819, 664; ^1^H NMR (500 MHz, CDCl_3_) δ: 7.94 (d, *J* = 8.5 Hz, 2H, Ar-H) 7.41 (d, *J* = 8.0 Hz, 2H, Ar-H), 7.34 (s, 1H, H-5), 6.20 (s, 1H, H-8), 5.99 (dd, *J* = 10.0, 1.0 Hz, 2H, OCH_2_O), 5.22 (d, *J* = 10.5 Hz, 1H, H-1), 4.94-4.97 (m, 1H, H-11), 4.20-4.27 (m, 1H, H-11), 3.98 (s, 3H, OCH_3_), 3.86-3.92 (m, 7H, H-3 and 2×OCH_3_), 3.48-3.51 (m, 1H, H-2), 2.47 (s, 3H, CH_3_); HRMS *m/z* calcd for C_29_H_26_O_10_NCl_2_S ([M+H]^+^) 650.0649, found 650.0630.

***Data for Vc:*** Yield = 59%, yellow solid, m.p. 111-112 °C, [α]^20^_D_ = 22 (c 2.6 mg/mL,CHCl_3_); IR cm^-1^ (KBr): 3091, 2934, 2855, 1780, 1625, 1549, 1480, 1392, 1242, 1019, 823, 678; ^1^H NMR (500 MHz, CDCl_3_) δ: 7.92 (d, *J* = 8.5 Hz, 2H, Ar-H), 7.76 (d, *J* = 8. 5 Hz, 2H, Ar-H), 7.30 (s, 1H, H-5), 6.22 (s, 1H, H-8), 6.00 (dd, *J* = 10.0, 1.5 Hz, 2H, OCH_2_O), 5.24 (d, *J* = 10.0 Hz, 1H, H-1), 4.92-4.95 (m, 1H, H-11), 4.23-4.25 (m, 1H, H-11), 3.98 (s, 3H, OCH_3_), 3.92 (s, 3H, OCH_3_), 3.80-3.89 (m, 4H, H-3 and OCH_3_), 3.48-3.52 (m, 1H, H-2); HRMS *m/z* calcd for C_28_H_23_O_10_NBrCl_2_S ([M+H]^+^) 713.9598, found 713.9576.

***Data for VIc:*** Yield = 48%, yellow solid, m.p. 131-132 °C, [α]^20^_D_ = 15 (c 2.6 mg/mL, CHCl_3_); IR cm^-1^ (KBr): 3091, 2931, 2852, 1780, 1619, 1573, 1482, 1390, 1193, 1105, 817, 603; ^1^H NMR (500 MHz, CDCl_3_) δ: 7.90 (d, *J* = 8.5 Hz, 2H, Ar-H), 7.76 (d, *J* = 8.5 Hz, 2H, Ar-H), 7.14 (s, 1H, H-5), 6.72 (s, 1H, H-8), 6.04 (s, 2H, OCH_2_O), 5.78 (s, 1H, H-6’), 5.09 (d, *J* = 2.0 Hz, 1H, H-1), 4.52-4.55 (m, 1H, H-11), 4.34 (d, *J* = 10.0 Hz, 1H, H-11), 3.91 (s, 3H, OCH_3_), 3.86-3.90 (m, 1H, H-3), 3.84 (s, 3H, OCH_3_), 3.44 (dd, *J* = 8.5, 2.5 Hz, 1H, H-2), 3.36 (s, 3H, OCH_3_); HRMS *m/z* calcd for C_28_H_24_O_10_NBr_2_S ([M+H]^+^) 723.9482, found 723.9470.
